# Can environmental improvement change the population distribution of walking?

**DOI:** 10.1136/jech-2016-208417

**Published:** 2017-05-12

**Authors:** Jenna Panter, David Ogilvie

**Affiliations:** Department of Medicine, MRC Epidemiology Unit and UKCRC Centre for Diet and Activity Research (CEDAR), School of Clinical, University of Cambridge, Cambridge, UK

**Keywords:** Epidemiological methods, Health inequalities, Neighborhood/place, PHYSICAL ACTIVITY, PUBLIC HEALTH

## Abstract

**Background:**

Few studies have explored the impact of environmental change on walking using controlled comparisons. Even fewer have examined whose behaviour changes and how. In a natural experimental study of new walking and cycling infrastructure, we explored changes in walking, identified groups who changed in similar ways and assessed whether exposure to the infrastructure was associated with trajectories of walking.

**Methods:**

1257 adults completed annual surveys assessing walking, sociodemographic and health characteristics and use of the infrastructure (2010–2012). Residential proximity to the new routes was assessed objectively. We used latent growth curve models to assess change in total walking, walking for recreation and for transport, used simple descriptive analysis and latent class analysis (LCA) to identify groups who changed in similar ways and examined factors associated with group membership using multinomial regression.

**Results:**

LCA identified five trajectories, characterised by consistently low levels; consistently high levels; decreases; short-lived increases; and sustained increases. Those with lower levels of education and lower incomes were more likely to show both short-lived and sustained increases in walking for transport. However, those with lower levels of education were less likely to take up walking. Proximity to the intervention was associated with both uptake of and short-lived increases in walking for transport.

**Conclusions:**

Environmental improvement encouraged the less active to take up walking for transport, as well as encouraging those who were already active to walk more. Further research should disentangle the role of socioeconomic characteristics in determining use of new environments and changes in walking.

## Introduction

Epidemiological evidence supports an association between physical activity and lower risk of diabetes, cardiovascular disease and mortality,^1^ ^2^ but many people remain insufficiently active.^3^ Public health advocacy increasingly focuses on everyday activities such as walking as a target for intervention. Walking can be incorporated into everyday life relatively easily and if performed at moderate pace meets the definition of moderate intensity activity.^4^ Modifying environments to make walking easier could produce widely distributed and sustained effects.^5–7^

Robust evidence on the impact of environmental changes on activity levels is beginning to emerge, but many existing studies have significant limitations in conceptualising and defining exposed populations^8^ and constructing controlled comparisons,^9^ or are limited by relatively short follow-up periods or imprecise measures of activity.^5^ ^6^ Some studies report no effect of environmental changes on time spent in activity,^10^ despite the fact that new or modified environments are well used. Others report a relatively small mean change in activity, which may mask substantial changes in some individuals.^11^ These observations beg the question whether these interventions have encouraged those who were already active to do more, or have encouraged the less active to take up new activity. Understanding how different population groups respond to interventions is essential for assessing their overall impacts on health and health inequalities, and for the design and targeting of future interventions. Adopting and maintaining health behaviours, such as walking, is a process that evolves over time^12^ and is therefore best understood using data from multiple time points.

Connect2 was a programme of engineering projects that aimed to make local walking and cycling journeys easier by constructing or improving routes at sites around the UK.^13^ The before-and-after evaluation of the Connect2 projects in Southampton, Kenilworth and Cardiff found that living closer to the new infrastructure was associated with increases in walking, cycling and overall physical activity at 2-year follow-up.^11^ Previous analysis^14^ concluded that the new routes were mostly used for walking, which therefore forms the behavioural focus for the more detailed analysis described in this paper. We aimed to describe changes in walking in the sample, identify groups of participants whose walking behaviour changed in similar ways and investigate the extent to which walking group membership differed by sociodemographic or health characteristics or exposure to the intervention.

## Methods: intervention

Connect2 projects were located at 79 sites in the UK. Each project included a core component, such as a bridge over a busy road, railway or river, together with the development or improvement of feeder routes. Interventions of this kind are often described by researchers as natural experiments, and this provided an opportunity to generate evidence about impacts. The independent iConnect study (http://www.iconnect.ac.uk) set out to measure and evaluate the travel, physical activity and carbon impacts of the Connect2 programme. Sites were selected for evaluation based on seven criteria, including implementation timetable and likelihood of measurable population impact.^13^ Three projects were selected for detailed evaluation: (1) Cardiff, where a traffic-free bridge was built over Cardiff Bay; (2) Kenilworth, where a traffic-free bridge was built over a busy trunk road; and (3) Southampton, where an informal riverside footpath was turned into a boardwalk.

### Participants and procedures

Detailed description of methods of the data collection has been reported elsewhere.^13^ Briefly, questionnaires were posted to 22 500 adults who were listed on the edited electoral register and lived within 5 km by road of the Connect2 projects in Kenilworth, Cardiff and Southampton in April 2010. In total, 3516 individuals returned the questionnaires at baseline and 1304 (37% of baseline) returned the follow-up questionnaires in April 2011 and April 2012.

## Measures

### Walking

As the correlates of walking for transport and recreation differed^15^ and double counting of activity seemed unlikely in this study,^16^ we examined total walking, as well as in walking for transport and for recreation which were assessed at both time points. Walking for transport was assessed using a 7-day recall instrument covering journeys made for commuting, on business, for study, for shopping and personal business, and for social activities. Participants reported the total weekly time spent walking for each purpose and these totals were summed. They also reported the total time spent walking for recreation in the past week using an adapted version of the short form of the International Physical Activity Questionnaire.^17^ Total time spent walking was the sum of the times spent walking for transport and for recreation. The test–retest reliability and convergent validity of our short IPAQ are comparable to those of other questionnaires, including the unmodified short IPAQ.^17^ Changes in total time spent walking, walking for transport and for recreation were computed (2012 minus 2010).

### Sociodemographic and health characteristics

Demographic (sex, age, ethnicity and presence of any child under 16 in the household), socioeconomic (highest educational level, annual household income and employment status) and health characteristics (height, weight, general health and presence of long-term illness or disability limiting daily activities) were self-reported in 2010. Height and weight were used to compute body mass index and assign weight status.^18^ Baseline characteristics were used as covariates in the analysis; these are more plausible determinants of behaviour than characteristics assessed at follow-up, and using multiple time-varying characteristics would have resulted in complex and unstable models.

### Exposure to and use of the intervention

As in our previous work, proximity to the Connect2 intervention was used to operationalise different degrees of exposure to make controlled comparisons in natural experimental studies. We assessed proximity using the shortest road network distance between each participant's home and the nearest Connect2 access point. Those living closer were deemed more highly exposed than those living further away. In 2011 and 2012, participants also reported if they had walked or cycled on their local Connect2 route for transport or recreation.

### Analysis

Only participants with complete data at all three time points were included in analysis. We excluded those who moved home, or who reported changes in walking of >15 hours/week in any one-year period (which might reflect misreporting 15 min as 15 hours). We used Mplus software V.7.11 for analysis 1 and 2 and Stata V.13.0 for analysis 3. In analysis 1 and 2, weekly minutes of walking were converted to hours to reduce the variance and improve the stability of the models. Further details are given in online supplementary additional file 1.

10.1136/jech-2016-208417.supp1 supplementary data

#### Analysis 1: describing average changes in the sample

We used latent growth curve models (LGCMs)^19^ to describe the average change in the sample over time and the between-person differences around the average by fitting unconditional models.

#### Analysis 2: identifying groups whose walking behaviour changed in similar ways

We used two methods to identify groups whose walking behaviour changed in similar ways, reclassifying those reporting <30 min per week as ‘minimal walking’ to reduce the noise of measurement error. We choose the 30 min/week threshold because this reflected a natural grouping in the data. First, we used a simple binary classification of time spent walking at each time point (0: ‘minimal’—<30 min vs 1: ‘meaningful’—30 min or more) and identified all possible patterns of change over time (eg, 0-1-1: those who took up behaviour and 1-0-0: those who gave up behaviour). Second, we used latent class analysis (LCA)^18^ to identify subpopulations of individuals that were not directly observed, but inferred from multiple observations. This technique has been applied to studying trajectories of obesity^20^ and physical activity,^21^ and the impact of interventions on weight loss^22^ and general health.^23^ In LCA we used weekly hours spent walking, allowing within-individual variation within classes through the use of the INTEGRATION function; we also used categorical assessments of <30 min/week, 30–150 min/week and >150 min/week, corresponding to recommended levels of physical activity.^1^ In both approaches, we began with a one-class model equivalent to the null hypothesis that all participants followed the same trajectory. We then tested increasing numbers of classes and estimated the probability of each participant belonging to each class.

#### Analysis 3: correlates of group membership

After identifying the optimal number of groups, participants were assigned to the group for which they had the highest probability of membership. We examined whether sociodemographic, health characteristics and exposure to the intervention were associated with groups derived from simple group classifications and LCA, using logistic and multinomial logistic regression models adjusted for age, sex and intervention site.

#### Sensitivity analyses

We repeated analyses 2 and 3 using raw values of time spent walking without the reclassification of <30 min/week walking as ‘minimal’.

## Results

### Study sample and participant characteristics

Of the 1304 participants who returned the questionnaires at all three time points, 1258 and 1266 provided data on walking for transport and for recreation, respectively, and had neither moved home nor reported an extreme change in walking. There were no major differences between participants included in and those excluded from the analysis, (defined above) and few differences between the sample used here and that used in the previous evaluation (table 1).^11^

**Table 1:** Sample characteristics of participants included in latent growth curve and class analysis

Variable	Category	Total walking per cent (N)	Walking for transport per cent (N)	Walking for recreation per cent (N)
Site	Southampton	27.5 (340)	27.5 (347)	27.6 (349)
	Cardiff	31.7 (391)	31.4 (395)	31.6 (400)
	Kenilworth	40.8 (503)	41.1 (518)	40.8 (517)
Sex	Female	55.0 (679)	55.1 (693)	55.3 (700)
	Male	45.0 (555)	44.9 (565)	44.7 (566)
Age (years) at baseline	18–34	8.8 (109)	8.8 (110)	9.0 (113)
	35–49	19.5 (241)	19.4 (244)	19.5 (247)
	50–64	35.4 (437)	35.2 (443)	35.1 (444)
	65–89	36.3 (446)	36.6 (460)	36.4 (461)
Ethnicity	White	967 (1193)	96.7 (1215)	96.7 (1222)
	Non-White	3.3 (40)	3.3 (41)	3.3 (42)
Any child under 16 in household	No	85.7 (1057)	86.0 (1081)	85.8 (1086)
	Yes	14.3 (177)	14.0 (177)	14.2 (180)
Highest educational level	Tertiary or higher	39.9 (492)	39.9 (502)	39.8 (505)
	Secondary school	33.4 (412)	32.9 (414)	32.8 (417)
	Lower than secondary	26.7 (330)	272 (342)	27.3 (34)
Annual household income	>£40 000	32.0 (391)	31.8 (396)	31.2 (395)
	£20 001–40 000	33.1 (405)	33.6 (419)	32.9 (417)
	≤£20 000	34.9 (426)	34.6 (431)	35 (442)
Employment Status	Working/ student	50.4 (622)	50.2 (632)	50.1 (635)
	Retired	42.2 (521)	42.5 (535)	42.2 (535)
	Home/sick	7.4 (91)	7.2 (91)	7.6 (96)
Any car in household	No	12.3 (151)	12.6 (158)	12.8 (162)
	Yes	87.7 (1083)	87.4 (1100)	87.2 (1104)
Weight status	Normal/underweight	48.4 (591)	48.7 (605)	48.9 (612)
	Overweight	36.6 (446)	36.6 (455)	36.5 (457)
	Obese	15.0 (183)	14.7 (183)	14.6 (183)
General health	Excellent/good	79.5 (9831	79.8 (1003)	79.6 (1007)
	Fair/poor	20.5 (252)	20.2 (253)	20.4 (258)
Long-term illness or disability that limits daily activities	No	74.6 (914)	74.6 (933)	74.4 (936)
	Yes	25.4 (312)	25.4 (316)	25.6 (322)
Proximity to core C2	Mean km(SD)	2.93 (1.30)	2.94 (1.30)	2.92 (1.29)
Use of Connect2*	No	70.0 (862)	88.9 (1115)	72.2 (911)
	Yes	30.0 (369)	11.1 (140)	27.8 (351)

Data in each column refer to those who were included in the analyses for total walking, walking for transport and walking for recreation.

*Use of Connect2 was matched to the outcome (ie, use of Connect2 for walking for recreation was modelled in the analysis of walking for recreation).

### Analysis 1: describing average changes

The median total time spent walking decreased slightly between 2010 and 2012 from 2.8 to 2.5 hours/week (p=0.35) and this was mostly explained by a decrease in walking for transport (see online supplementary additional file A2, table SA1), but there were some large within-person changes (see online supplementary additional file A2 and figure SA1). In simple LGCMs, we fixed the slope factor loadings at 0, 1 and 2 for 2010, 2011 and 2012. We tested whether the average population change was linear by freeing the slope parameter for 2011. If change was constant between 2010 and 2012, the estimated values for 2011 should have been close to 1, matching a linear change over a 1-year period. The freed slope parameter for total walking was 1.3 (SE: 2.57), indicating that the average change between 2010 and 2011 was slightly greater than that between 2011 and 2012. The model fit and parameters for LGCMs with fixed and freed slopes were generally similar (those for total walking are shown in table 2). The mean slope of the growth lines showed no significant overall change over time; however, individuals differed in their baseline levels of walking and their rate of change over time (as indicated by statistically significant intercept and slope variances, both p<0.001). Participants with higher levels of walking at baseline were more likely to report a decrease over time, as indicated by the negative intercept–slope correlation.

**Table 2:** Latent growth curve models for total walking

	With slope estimates for 2010, 2011 and 2012 fixed		With slope estimate for 2011 allowed to vary	
Parameters of the growth curve	Coefficient	SE	Coefficient	SE
Intercept mean	4.07***	0.12	4.08***	0.12
Intercept variance	15.14***	0.99	16.50	15.33
Slope mean	−0.02	0.06	−0.02	0.06
Slope variance	1.61***	0.41	1.68	2.47
Intercept–slope correlation	−0.252		−0.192	
Model fit statistics				
RMSEA†	0.001		0.001	
χ^2^	0.057		1597.6	
p	0.8118		0.001	
CFI‡	1.000		1.000	
TLI	1.000		1.000	
SRMR	0.001		0.001	

For further information on the model fit statistics, see online supplementary additional file 1.

***p<0.001, **p<0.01, *p<0.05, NS p>0.05.

†Should be <0.08.

‡Should be close to 1.

AIC, Akaike Information Criterion; CFI, comparative fit index; RMSEA, root mean square error of approximation; NS, not significant; SRMR, standardised root mean square residual; TLI, Tucker-Lewis index.

### Analysis 2: identifying groups

Simple binary descriptive analysis illustrated that those who reported some walking at all time points (39–42%) formed the largest group, whereas smaller numbers of people either took up walking from scratch or gave it up (<16%; online supplementary additional file 2, figure SA2).

In LCA, for all measures of walking the models with four and five classes produced the best improvements in model fit statistics, entropy and class size (table 3). We chose the five-class model because it better distinguished groups reporting lower levels of activity, which represented a large number of participants. Results using the categorical measures of change, and the unreclassified continuous measures of time by way of sensitivity analysis are shown in online supplementary additional file 2, table SA2.

**Table 3:** Results of longitudinal latent class analysis for total walking and walking for transport and for recreation

	Total time spent walking				Time spent walking for recreation					Time spent walking for transport				
	Two classes	Three classes	Four classes	Five classes	Two classes	Three classes	Four classes	Five classes	Six classes	Two classes	Three classes	Four classes	Five classes	Six classes
Sequential model comparisons	2 v 1	3 v 2	4 v 3	5 v 4	2 v 1	3 v 2	4 v 3	5 v 4	6 v 5	2 v 1	3 v 2	4 v 3	5 v 4	6 v 5
Log likelihood values for T classes	−10931.2	−10156.5 H0 Loglikelihood Value -10156.506	−9853.1	−9675.2	−9123.4	−8858.4	−8666.8	−8570.0	−8483.6	−8906.2	−8643.5	−8453.5	−8278.2	−8163.7
−2 difference in log likelihood	1549.4	606.8	201.4	154.1	1509.30	530.03	383.06	193.52	172.98	1157.37	525.24	380.02	350.52	229.13
Lo-Mendell-Rubin adjusted LRT value	1496.8	586.3	194.6	148.9	1458.28	512.11	370.11	186.98	167.13	1118.21	525.24	367.161	338.67	221.38
Lo-Mendell-Rubin adjusted LRT p value	0.001	0.001	0.001	0.144	0.001	0.030	0.048	0.1417	0.159	0.036	0.466	0.0512	0.0476	0.420
Bootstrap LRT p value	0.001	0.001	0.010	0.153	0.001	0.001	0.001	0.001	0.001	0.001	0.472	0.001	0.001	0.001
Information criterion														
AIC	20333.0	19734.1	19540.7	19394.5	18266.7	17744.7	17369.6	17184.1	17019.1	17832.3	17315.1	16943.0	16600.5	16379.4
BIC	20384.2	19805.8	19632.8	19507.2	18318.2	17816.8	17462.3	17297.3	17153.0	17883.7	17387.0	17035.6	16713.6	16513.0
Sample size adjusted BIC	20352.5	19761.3	19575.7	19437.3	18286.4	17772.3	17405.1	17227.5	17070.4	17851.9	17342.6	16978.4	16643.7	16430.4
Entropy	0.93	0.912	0.912	0.870	0.946	0.945	0.950	0.955	0.954	0.967	0.945	0.937	0.955	0.946
Smallest class size (%)	14.6	5.0	4.8	4.1	13.9	2.6	2.4	2.6	3.17	7.3	1.9	0.79	0.64	0.55

AIC, BIC difference in the number of parameters for all models is 4.

AIC, Akaike Information Criterion BIC, Bayesian Information Criterion; LRT, likelihood ratio test.

**Figure 1:**
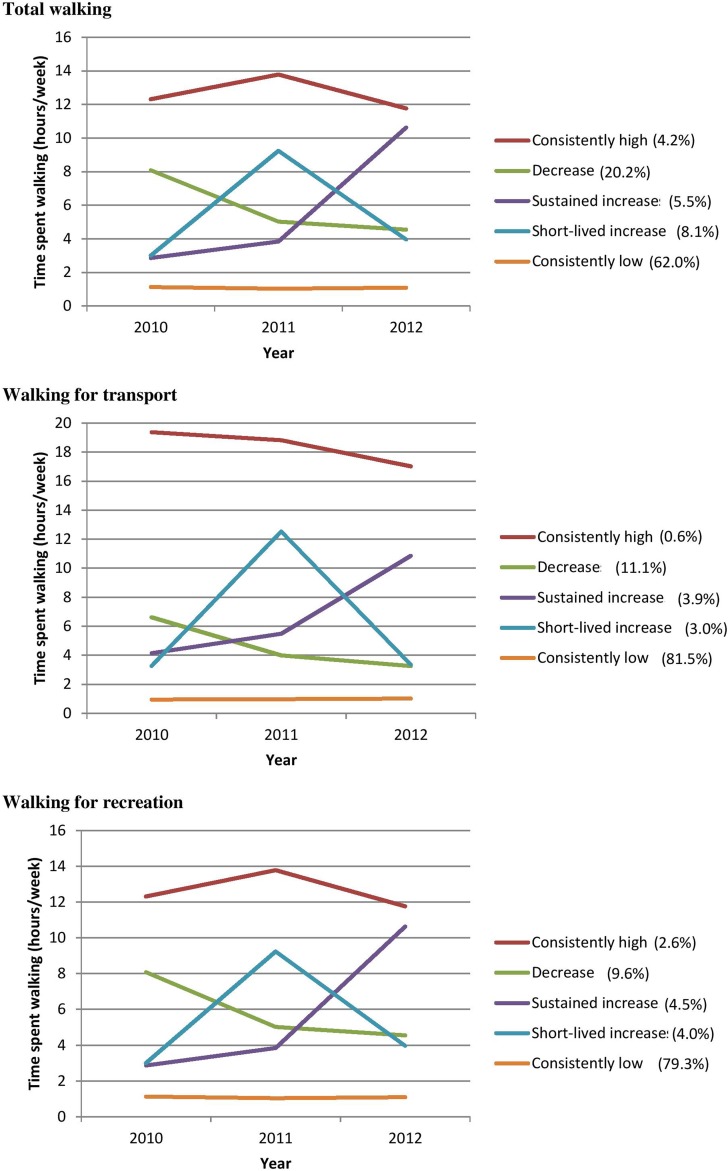
Estimated growth curves for five-class longitudinal latent class analysis model.

The five distinct trajectories were characterised by consistently low, consistently high, sustained decreases in, short-lived increases in and sustained increases in levels of walking. Estimated growth curves for each class are shown in figure 1. Across the three outcomes, the shapes of trajectories were similar and the group with consistently low levels of walking was always the largest. Membership of classes based on walking for transport and walking for recreation was strongly related (χ^2^=165.4, p<0.001); but the separation of the classes was good, as shown by the entropy scores and the correlation between assigned and average classes (see online supplementary additional file 2, table SA3 and table SA4).

### Analysis 3: correlates of group membership

We focused on understanding the characteristics of those who demonstrated either short-lived or sustained increases in walking for transport or for recreation (identified in LCA), and of those who took up either behaviour, as these outcomes are associated with potential health gain (and the trajectories for total time spent walking were very similar). For the analysis of increases in walking, participants whose levels of walking remained consistently low formed the reference group (total n: walking for recreation=1115; walking for transport=1105). For the analysis of uptake of walking, those who never walked formed the reference group (total n: 383, 394 respectively). Sample sizes are given in online supplementary additional file 2, table SA5.

Both short-lived and sustained increases in, and uptake of, walking were socioeconomically patterned (table 4). Compared to those who reported consistently low levels of walking for transport, participants who reported short-lived or sustained increases were more likely to have lower household incomes, lower levels of education and no access to a car. However, the findings for uptake of walking were the opposite. Those with lower levels of education were less likely to take up walking for recreation or transport, and those with lower incomes were less likely to take up walking for recreation. Those who were obese, reported fair or poor general health, or had a limiting long-term condition were also less likely to take up walking.

**Table 4:** Multinomial logistic regression models examining the associations between changes in walking and sociodemographic and health characteristics, and intervention exposure and use

Outcome	Walking for transport			Walking for recreation RRR (95% CI)		
Baseline characteristics	Short-lived increase† RRR (95% CI)	Sustained increase† RRR (95% CI)	Uptake‡ OR (95% CI)	Short-lived increase† RRR (95% CI)	Sustained increase† RRR (95% CI)	Uptake‡ OR (95% CI)
*Demographic*						
Site (Ref: Southampton)	1.0	1.0	1.0	1.0	1.0	1.0
Cardiff	0.69 (0.32 to 1.48)	0.98 (0.47 to 2.02)	0.94 (0.42 to 2.13)	1.73 (0.76 to 3.96)	0.58 (0.26 to 1.29)	1.11 (0.52 to 2.34)
Kenilworth	0.37 (0.16 to 0.87)	0.74 (0.36 to 1.53)	2.18 (1.02 to 4.66)	1.56 (0.70 to 3.50)	1.34 (0.70 to 2.56)	1.34 (0.66 to 2.73)
Sex (Ref: Female)	1.0	1.0	1.0	1.0	1.0	1.0
Male	0.76 (0.38 to 1.49)	0.95 (0.53 to 1.71)	0.89 (0.47 to 1.72)	1.23 (0.68 to 2.23)	0.70 (0.39 to 1.25)	0.91 (0.50 to 1.66)
Age (Ref: 18–34) years)	1.0	1.0	1.0*	1.0	1.0*	1.0*
35–49	0.39 (0.10 to 1.50)	1.25 (0.39 to 4.06)	0.87 (0.18 to 4.18)	0.71 (0.20 to 2.59)	**0.81 (0.31 to 2.12)**	1.51 (0.38 to 6.00)
50–64	0.82 (0.29 to 2.34)	0.91 (0.29 to 2.84)	1.42 (0.32 to 6.23)	1.34 (0.44 to 4.07)	**0.77 (0.31 to 1.89)**	2.13 (0.57 to 7.95)
65–89	0.71 (0.24 to 2.06)	1.09 (0.36 to 3.33)	0.39 (0.09 to 1.60)	1.01 (0.33 to 3.12)	**0.42 (0.16 to 1.11)**	0.55 (0.16 to 1.96)
Ethnicity (Ref: white)	1.0	1.0	1.0	1.0	1.0	1.0
Non-white	1.54 (0.33 to 7.29)	2.21 (0.62 to 7.87)	0.79 (0.27 to 2.26)	1.53 (0.34 to 6.83)	1.97 (0.64 to 6.07) to 5.94)	1.30 (0.51 to 3.37)
Children (Ref: none) Any	1.0 0.39 (0.08 to 1.83)	1.0 1.09 (0.42 to 2.82)	1.0 1.11 (0.54 to 2.28)	1.0 1.42 (0.51 to 3.89)	1.0 0.64 (0.26 to 1.59)	1.0 1.28 (0.65 to 2.54)
*Socioeconomic*						
Educational level (Ref: tertiary)	1.0*	1.0**	1.0*	1.0	1.0	1.0***
Secondary school or higher	**1.61 (0.72 to 3.58)**	**1.15 (0.52 to 2.58)**	**0.50 (0.31 to 0.82)**	0.59 (0.29 to 1.21)	1.20 (0.62 to 2.33)	**0.66 (0.41 to 1.07)**
Lower than secondary	**1.36 (0.54 to 3.44)**	**3.21 (1.48 to 6.94)**	**0.76 (0.45 to 1.29)**	0.59 (0.26 to 1.31)	1.55 (0.73 to 3.28)	**0.34 (0.20 to 0.60)**
Car ownership (Ref: any car)	1.0***	1.0***	1.0	1.0	1.0	1.0
No car	**5.35 (2.58 to 11.08)**	**4.77 (2.42 to 9.42)**	1.24 (0.54 to 2.85)	0.51 (0.15 to 1.70)	1.18 (0.51 to 2.72)	0.68 (0.37 to 1.25)
Annual household income, £ (Ref: >40 000)	1.0**	1.0**	1.0	1.0	1.0	1.0*
20 001–40 000	**1.92 (0.69 to 5.33)**	**2.41 (1.00 to 5.82)**	1.17 (0.71 to 1.93)	0.59 (0.28 to 1.26)	1.11 (0.58 to 2.12)	**0.63 (0.37 to 1.08)**
≤20 000	**3.43 (1.26 to 9.33)**	**3.79 (1.55 to 9.26)**	0.74 (0.42 to 1.31)	0.63 (0.29 to 1.40)	0.58 (0.25 to 1.33)	**0.49 (0.28 to 0.85)**
Employment status (Ref: working/student)	1.0	1.0	1.0	1.0	1.0	1.0
Retired	2.59 (0.96 to 6.95)	2.85 (1.10 to 7.40)	1.10 (0.60 to 2.00)	1.60 (0.68 to 3.75)	1.59 (0.68 to 3.76)	0.67 (0.33 to 1.35)
Unemployed/Other/Sick	0.84 (0.19 to 3.74)	1.94 (0.70 to 5.38)	1.15 (0.46 to 2.85)	1.05 (0.30 to 3.63)	0.21 (0.03 to 1.59)	0.74 (0.33 to 1.65)
*Health*						
Weight status (Ref: normal)	1.0	1.0	1.0*	1.0*	1.0	1.0*
Overweigh	1.16 (0.58 to 2.33)	0.66 (0.33 to 1.31)	**0.88 (0.57 to 1.37)**	0.56 (0.29 to 1.09)	0.59 (0.30 to 1.13)	**0.90 (0.58 to 1.41)**
Obese	0.32 (0.07 to 1.42)	1.10 (0.50 to 2.41)	**0.44 (0.24 to 0.80)**	0.31 (0.09 to 1.03)	0.70 (0.30 to 1.63)	**0.40 (0.22 to 0.74)**
General health (Ref: excellent-good)	1.0	1.0	1.0***	1.0	1.0	**1.0**
Fair-poor	1.13 (0.52 to 2.48)	0.84 (0.39 to 1.78)	**0.36 (0.21 to 0.61)**	0.61 (0.27 to 1.40)	0.60 (0.27 to 1.37)	**0.42 (0.26 to 0.68)**
Limiting long-term condition (Ref: no) Yes	1.0 0.97 (0.43 to 2.18)	1.0 0.60 (0.27 to 1.32)	1.0** 0.50 (0.31 to 0.81)	1.0 1.12 (0.55 to 2.27)	1.0 1.13 (0.55 to 2.34)	1.0*** 0.32 (0.19 to 0.54)
*Exposure to C2*						
Per km closer to core C2	1.40 (1.04 to 1.89)*	1.13 (0.88 to 1.45)	**1.21 (1.03 to 1.42)‡**	1.03 (0.80 to 1.32)	1.07 (0.85 to 1.35)	1.14 (0.96 to 1.37)
*Use of Connect2* (Ref: Not) used)	1.0**	1.0*	1.00***	1.0*	1.0***	1.0***
Any¶	**3.69 (1.67 to 8.18)**	**2.56 (1.21 to 5.44)**	**2.87 (1.31 to 6.30)**	**2.13 (1.14 to 4.00)**	**3.00 (1.66 to 5.42)**	**3.44 (2.02 to 5.84)**

*p<0.05, **p<0.01, ***p<0.001.

†Reference trajectory was that of participants who reported consistently low levels of walking.

‡Reference trajectory was that of participants who never reported walking.

p Values are tests for trend in the case of variables with three or more categories.

¶Use of Connect2 was measured in 2012 and matched to the outcome (ie, use of Connect2 for walking for recreation was modelled in the analysis of walking for recreation).These models are adjusted for age, sex and site.

RRR, relative risk ratio.

In adjusted multivariable models, participants living closer to the Connect2 routes were more likely than those living further away to show a short-lived increase in walking for transport or to take it up. Use of Connect2 was also associated with uptake of, and short-lived and sustained increases in, walking; these associations were strongest for short-lived increases in walking for transport and uptake of walking for recreation. Results for the sensitivity analyses were similar (see online supplementary additional file 2, table SA6).

## Discussion

### Principal findings

We employed simple descriptive and LCA approaches to identify groups whose walking behaviour changed in similar ways over 2 years using data from a natural experimental study of new transport infrastructure. Five distinct trajectories were identified from LCA, characterised by consistently low, consistently high, sustained decreases in, short-lived increases in and sustained increases in levels of walking. There were socioeconomic differences between the groups identified with these trajectories. Residential proximity to the intervention, which was not socioeconomically patterned, was independently associated with both short-lived increases in and uptake of walking for transport.

### Strengths and limitations

We sought to generate more robust evidence on who benefits from environmental interventions, as recommended in previous guidance, through population-based sampling, the use of three intervention sites and use of controlled comparisons.^5^ ^6^ In contrast to simpler methods of assessing change, for example, by calculating absolute change^11^ or categorising individuals into groups whose activity increased or decreased,^24^ our analysis more fully exploited the longitudinal nature of the data, including the sequence and timing of changes. We used complementary approaches to understand who changed and how, using a combination of theoretically important groupings as well as those that emerged in the course of analysis. We used three repeated measures which is the minimum number necessary for LCA,^19^ but four or more would have enabled us to explore the possibility of a wider range of trajectories, including fitting piecewise models.

Measures of both exposure and outcome were specific to the intervention, including purpose-specific measures of use of the infrastructure. Although the iConnect study had a comparatively low response rate, this is not unusual in natural experimental studies of this kind.^25^ The cohort was slightly older and healthier than the local populations,^11^ but comparable in many other characteristics, although our sample cannot be assumed to representative. Further studies need to be undertaken to assess the effects in other populations and contexts. Changes in walking may have occurred for a range of reasons, some unrelated to the intervention. Although we excluded people who moved home, we had limited data on other life events, such as pregnancy, which might have influenced levels of walking. Future research might further explore the role of such time-varying characteristics.

### Patterns and trajectories in walking

Changes in physical activity are often short-term, and little is known about the sustainability of changes in everyday activities such as walking. In this sample, we observed a small average decline in walking over time. On average, those with higher levels of walking at baseline were more likely to report declines and four of the five trajectories we identified were characterised by high initial levels of walking. As the median time spent walking in the sample was relatively high, this may reflect regression to the mean^26^ whereby extreme scores on one measure become less extreme over time.

We also found evidence that change in time spent walking was not linear, in that the average change between 2010 and 2011 was greater than that between 2011 and 2012. Our LCA did not identify a group corresponding to this pattern of change, which suggests that the overall average pattern conceals several divergent underlying groups. In fact in the group who demonstrated a sustained increase in walking we observed the opposite pattern, with the larger increase occurring in the second interval (between 2011 and 2012). The fact that exposure to the intervention was associated with sustained increases in walking supports the findings of other studies^11^ ^27^ ^28^ which suggest that the effect of environmental interventions on physical activity patterns may take some time to emerge.

Previous research has indicated that walking is socioeconomically patterned,^29^ and we found further support for this. Measures of socioeconomic status were associated with changes in walking for transport and for recreation, in opposite directions; these associations were explained by baseline differences in walking levels. The groups who demonstrated increases in walking for transport started from relatively high baseline levels, which may reflect limited choice in their transport options. Unsurprisingly, therefore, these trajectories were associated with lower levels of education and income and with lack of access to a car. In our analysis of uptake of walking, in contrast, we compared groups who reported minimal walking at baseline but differed in their subsequent trajectories. Here, we found that participants with lower levels of education or income were less likely to take up walking during the study, a pattern consistent with the literature on inequalities in walking.^29^

### Implications and future research

People who were more exposed to the new infrastructure were more likely to take up, or to demonstrate a short-lived increase in, walking for transport and exposure to the intervention was not socioeconomically patterned. Interventions like Connect2 might, therefore, be concluded to encourage initiation of walking. The fact that these routes were visible and are described as ‘living landmarks’ may in part explain the success of Connect2.^30^ Although we also treated ‘use’ of the infrastructure as an additional measure of exposure, this entails a degree of circularity because use of the infrastructure necessarily involves the behavioural outcome of interest.^31^ In this sense our finding that use was consistently associated with increases in walking, whereas proximity was associated with short-term but not with sustained increases in walking, raises the possibility that while environmental improvements of this kind may encourage initiation, they may be insufficient for promoting maintenance of new behaviours without addressing other factors such as social support. This contrasts with longitudinal evidence from an Australian observational study.^32^ Given the socioeconomic inequalities in walking and the characteristics of early and late adopters of such interventions,^14^ one priority for future research should be to disentangle the pathways by which interventions work (or do not work) for different groups.

**What is already known on this subject**

- Modifying environments to make walking easier could produce sustained effects that are widely distributed in the population. There is now a growing set of evaluative studies assessing the impact of changes to the environment on walking.
- It is unknown whether these interventions have encouraged those who were already active to do more or whether the inactive are taking up activity.
- Understanding which population groups increase their physical activity is essential for assessing the health impacts of interventions and the impact on health inequalities.

**What this study adds**

- We identified five groups who changed their walking in similar ways and we found that membership of these groups was socioeconomically patterned.
- Proximity to the intervention was associated with short-lived increases in and uptake of walking for transport. These measures of proximity were not socioeconomically patterned.
- This suggests that supportive environments may help initiate, but not maintain behaviour change.
