# Supplementary material for: Can environmental improvement change the population distribution of walking?
Source: J Epidemiol Community Health. 2017 May 12;71(6):528–35. doi: 10.1136/jech-2016-208417 (PMC5484036; doi:10.1136/jech-2016-208417)
Supplement: supplementary data [file jech-2016-208417supp001.pdf]

Additional file 1. Further methodological information

Analysis 1: Describing average changes in the sample

We used a latent growth modelling framework to capture the average change over time and the between-person differences around the average by fitting unconditional latent growth models (example shown in Figure A1). We used 2000 initial random starts and 200 final stage optimisations to fit each model to ensure that a true (rather than a local) maximum likelihood solution had been reached. Overall model fit was determined using the recommendations of Bentler.[1] We used the Tucker Lewis Index (TFI) and the Comparative Fit Index (CFI) [1], with values >0.95 indicating reasonable model fit and values >0.90 indicating a plausible model; and the Standardized Root Mean Residual (SRMR), [2] with values <0.05 indicating reasonable model fit and values <0.08 indicating a plausible model.

Figure A1: Examples of unconditional latent growth curve models fitted in Mplus

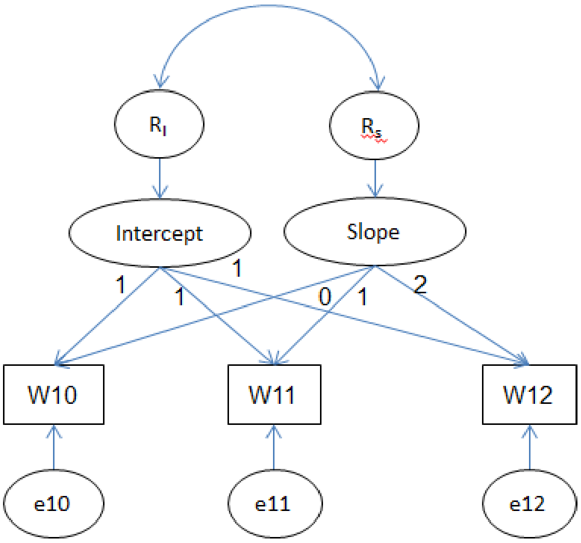

Three observed variables: W10: reported weekly time spent walking in 2010; W11: reported weekly time spent walking in 2011; W12: reported weekly time spent walking in 2012.
Two latent variables: intercept and slope. The intercept is identified by the constant loadings of 1 going to each walking time (constant effect). The slopes are fixed at 0, 1 and 2 representing the yearly intervals between measurements.
Intercept and slope have individual variance (RI and RS respectively). e terms represent individual error terms for reported time spent walking.

Analysis 2: Identifying classes

The standard Likelihood ratio test (LRT) is not valid when using the latent class approaches, so we used the Lo-Mendell-Rubin adjusted LRT and Bayesian Information Criterion (BIC). The Lo-Mendell-Rubin adjusted LRT tests the model that has T classes against the model with T−1 classes, with a significant *P* value indicating that the T-class model provides a better fit to the data. Smaller AIC and BIC values indicate a better fit. Models that best combine goodness of fit and parsimony are indicated by minimum values of the information criteria. The entropy, relative sample sizes for each class, and meaningful interpretation of the classes were also considered. Entropy is a summary statistic based on the membership probabilities that evaluates the quality of the classification in terms of the separation of the latent classes. Values of entropy range from 0 to 1, with scores close to 1 indicating clear classifications.[3]

References:

1. Bentler PM. Comparative fit indices in structural models. Psychological Bulletin 1990;107:238-246.
2. Hu L, Bentler PM. Cutoff criteria for fit indexes in covariance structure analysis: Conventional criteria versus new alternatives, Structural Equation Modeling: A Multidisciplinary Journal 1999;6:1-55.
3. Muthén L, Muthén B. Mplus User's Guide. Seventh Edition. Los Angeles, CA: Muthén & Muthén, 2012.

<u>Additional file 2: Additional results</u>

Table A1: Average weekly time spent walking in 2010, 2011 and 2012

	**2010**	**2011**	**2012**
**Total walking**			
Median (IQR) h/week	2.83 (0.75, 6.0)	2.50 (0.63, 6.0)	2.50 (0, 5.67)
**Walking for transport**			
Median (IQR) h/week	0.92 (0, 2.92)	0.75 (0, 2.50)	0.75 (0.0, 2.50)
**Walking for recreation**			
Median (IQR) h/week	1.0 (0.0, 3.0)	1.0 (0.0, 3.0)	1.0 (0.0, 3.0)

Figure A1: Individual profiles of weekly total walking, walking for transport and for recreation between 2010 and 2012

A. Total walking

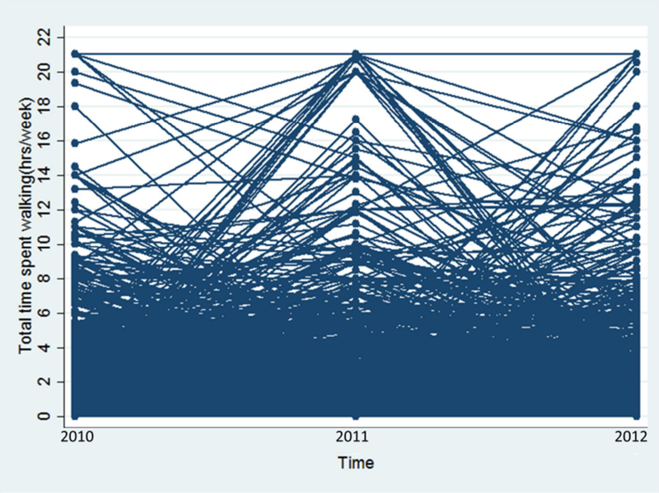

B. Walking for transport

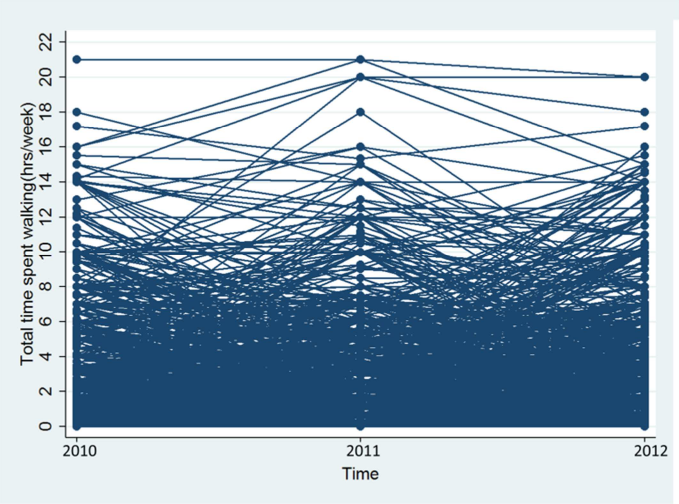

C. Walking for recreation

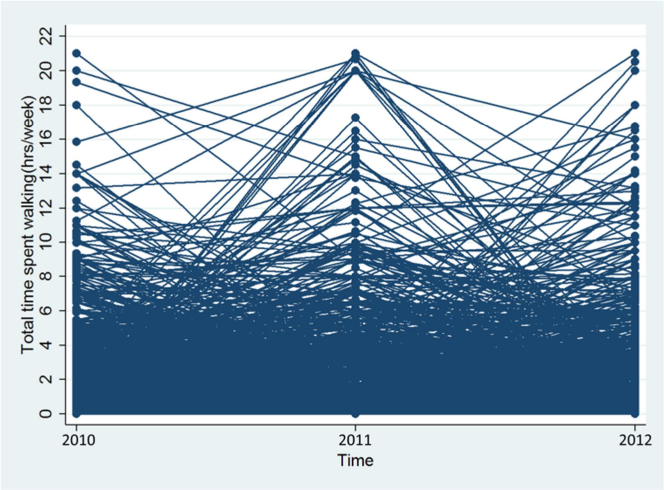

Figure A2: Patterns of change in total walking, walking for recreation and walking for transport over two years

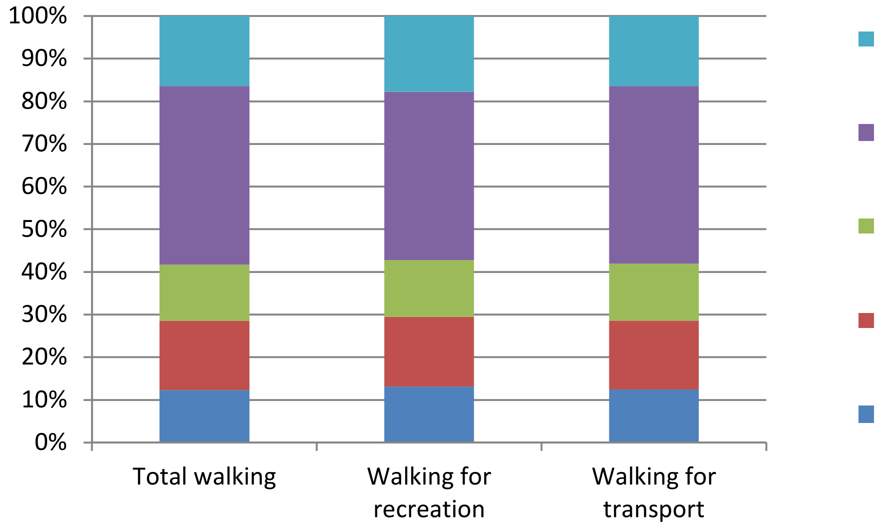

Description	2010	2011	2012
Never reported any walking	0	0	0
Always reported some walking	1	1	1
Took up walking	0	1	1
	0	0	1
Gave up walking	1	0	0
	1	1	0
Unstable levels of walking	0	1	0
	1	0	1

0=Minimal, 1=Meaningful

Table A2: Results of longitudinal latent class analysis using categorical measures and unreclassified continuous measures of walking

	**Categorical measures**				**Unreclassified continuous measures**				
	1 class	2 class	3 class	4 class	1 class	2 class	3 class	4 class	5 class
**Total walking**									
Likelihood	-3759.6	-3351.53	-3283.5	-3282.3	-10917.20	-10138.72	-9833.87	-9731.538	-9654.516
Class distribution [%]									
Class 1	100%	43.90%	46.60%	1.20%	100%	85.5%	21.2%	71.79%	5.5%
Class 2		56.10%	42.70%	10.90%		14.5%	73.8%	17.62%	8.00%
Class 3			10.70%	42.20%			5.0%	5.74%	62.54%
Class 4				45.70%				4.85%	4.18%
Class 5									19.8%
BIC	7540.61	6715.78	6631.1	6650.0	21877.12	20348.66	19767.42	19591.24	19507.16
Entropy	-	0.737	0.731	0.770	-	0.93	0.91	0.91	0.87
n	1237	1237	1237	1237	1237	1237	1237	1237	1237
**Walking for recreation**									
Likelihood	-4152.62	-3806.47	-3689.8	*	-9857.67	-9104.17	-8838.27	-8645.50	-8546.86
Class distribution [%]									
Class 1	100%	45.50%	38.90%		100%	86.0%	16.4%	79.8%	3.9%
Class 2		54.50%	39.70%			14.0%	81.1%	11.5%	9.6%
Class 3			21.40%				2.5%	6.2%	79.4%
Class 4								2.5%	4.5%
Class 5									2.6%
BIC	8348.12	7705.8	7522.54		19758.23	18279.81	17776.59	17419.64	17250.9
Entropy	-	0.675	0.693		-	0.946	0.945	0.951	0.955
n	1270	1270	1270		1270	1270	1270	1270	1270
**Walking for transport**									
Likelihood	-4117.11	-3768.09	-3706.59	*	-9450.75	-8858.87	-8589.19	-8389.86	
Class distribution [%]					100%	7.12	12.4	5.7	3.9
Class 1	100%	55.90%	38.20%			92.8	86.2	7.2	10.8
Class 2		44.10%	36.70%				1.4	85.8	81.7
Class 3			25.10%					1.30	2.9
Class 4									0.6
Class 5									
BIC	8277.07	7629.01	7556		18944.35	17789.15	17278.35	16908.24	
Entropy	-	0.703	0.617		-	0.969	0.950	0.964	0.957
n	1262	1262	123		1262	1262	1262	1262	1262

*Models would not converge due to small sample size and overfitting. - No entropy reported for a one-class model.

Correlation of and separation between class membership for walking for transport and for recreation

Table A3: Class membership for walking for transport and walking for recreation

	**Walking for recreation**					
**Walking for transport**	Consistently low levels	Consistently high levels	Sustained increases	Short-lived increases	Decreases	**Total**
Consistently low levels	869 (85.2)	19 (1.9)	33 (3.2)	22 (2.2)	77 (7.5)	1,020
Consistently high levels	2 (40)	1 (20)	0 (0)	0 (0)	2 (40)	5
Sustained increases	24 (50)	3 (6.2)	7 (14.6)	7 (14.6)	7 (14.6)	48
Short-lived increases	16 (43.2)	5 (13.5)	2 (5.4)	10 (27.1)	4 (10.8)	37
Decreases	91 (66.91)	5 (3.7)	8 (5.9)	6 (4.4)	26 (19.1)	136
**Total**	1,002	33	50	45	116	1,246

Numbers of participants (row percentage) classified in each category

Table A4. Comparison between assigned and average latent classes for walking for transport and for recreation

Assigned latent trajectory class	Average latent class assignment probability*				
**Walking for transport**	Consistently low levels	Consistently high levels	Sustained increases	Short-lived increases	Decreases
Consistently low levels	**0.901**	0.015	0.08	0.001	0.004
Consistently high levels	0.042	**0.933**	0.021	0.001	0.004
Sustained increases	0.015	0.001	**0.984**	0.001	0.001
Short-lived increases	0.001	0.001	0.001	**1.0**	0.001
Decreases	0.016	0.006	0.006	0.001	**0.972**
**Walking for recreation**	Consistently low levels	Consistently high levels	Sustained increases	Short-lived increases	Decreases
Consistently low levels	**0.986**	0.003	0.007	0.004	0.001
Consistently high levels	0.03	**0.912**	0.03	0.028	0.001
Sustained increases	0.032	0.027	**0.912**	0.028	0.001
Short-lived increases	0.028	0.035	0.035	**0.898**	0.005
Decreases	0.001	0.004	0.014	0.008	**0.973**

*Respondents were assigned to the latent trajectory classes for which the posterior probability of latent class membership was highest. Accuracy can be judged from the high diagonal and low off-diagonal elements in the assignment matrix.

Table A5: Sample sizes for group membership according to latent classes and simple descriptive classifications

		**Walking for recreation**	**Walking for transport**
Latent classes	Consistently low	1016	1020
	Short-lived increases	46	37
	Sustained increases	53	48
	Total sample	1115	1105
Simple descriptive classification	0 Never	252	264
	1 Takes up	170	174
	Total sample	422	438

Table A6: Logistic regression models of correlates of uptake of walking without reclassification (sensitivity analysis)

**Baseline characteristics**	**Uptake of any walking OR (95% CI)**	**Uptake of walking for transport OR (95% CI)**	**Uptake of walking for recreation OR (95% CI)**
**Demographic**			
Ethnicity (Ref: white)	1.0	1.0	1.0
Non-white	1.67 (0.23, 12.00)	1.39 (0.54, 3.59)	1.30 (0.51, 3.37)
Children in the household (Ref: none)	1.0	1.0	
Any	0.98 (0.24, 4.04)	1.10 (0.55, 2.22)	1.28 (0.65, 2.54)
**Socio-economic**			
Educational level (Ref: Tertiary or equivalent)	1.0	**1.0*** **	1.0***
Secondary school or higher	0.49 (0.21, 1.12)	**0.63 (0.38, 1.02)**	**0.66 (0.41, 1.07)**
Lower than secondary	0.48 (0.20, 1.14)	**0.32 (0.18, 0.57)**	**0.34 (0.20, 0.60)**
Car ownership in the household (Ref: no car)	1.0	**1.0*** **	1.0
No car	1.26 (0.74, 2.15)	**2.71 (1.39, 5.28)**	0.68 (0.37, 1.25)
Annual household income, £ (Ref: > 40 000)	1.0	**1.0***	**1.0***
20 001–40 000	0.62 (0.24, 1.63)	**0.73 (0.42, 1.26)**	**0.63 (0.37, 1.08)**
≤ 20 000	0.43 (0.16, 1.10)	**0.49 (0.27, 0.87)**	**0.49 (0.28, 0.85)**
Employment status (Ref: working/ student)	1.0	1.0	1.0
Retired	0.95 (0.34, 2.65)	0.73 (0.36, 1.49)	0.67 (0.33, 1.35)
Unemployed/Other/Sick	1.84 (0.39, 8.63)	0.76 (0.34, 1.71)	0.74 (0.33, 1.65)
**Health**			
Weight status (Ref: normal)	1.0	**1.0*** **	**1.0***
Overweight	1.33 (0.63, 2.83)	**0.91 (0.57, 1.44)**	**0.90 (0.58, 1.41)**
Obese	0.59 (0.24, 1.44)	**0.38 (0.20, 0.70)**	**0.40 (0.22, 0.74)**
General health (Ref: excellent-good)	**1.0*** **	**1.0*** **	**1.0**
Fair-poor	**0.25 (0.12, 0.52)**	**0.42 (0.26, 0.68)**	**0.42 (0.26, 0.68)**
Limiting long-term condition (Ref: no)	1.0	1.0***	1.0***
Yes	0.53 (0.25, 1.11)	**0.31 (0.19, 0.53)**	**0.32 (0.19, 0.54)**
**Exposure to C2**			
per Kilometre Closer to core C2	1.26 (0.93, 1.70)	**1.21 (1.00, 1.45)***	1.14 (0.96, 1.37)
**Use of Connect2 (Reference: Never)**	1.0	**1.0*** **	**1.0*** **
Any	**2.61 (1.19, 5.71)***	**2.80 (1.78, 4.41)*** **	**3.44 (2.02, 5.84)** **

*Adjusted for site, age and sex. *p<0.05,**p<0.01,***p<0.001; for categorical variables, tests for heterogeneity were used.
